# Thermally controlled microfluidic back pressure regulator

**DOI:** 10.1038/s41598-021-04320-6

**Published:** 2022-01-12

**Authors:** Karolina Svensson, Simon Södergren, Klas Hjort

**Affiliations:** Microsystems Technology Division, Centre of Natural Hazard and Disaster Science (CNDS), Uppsala University, Box 35, 751 03 Uppsala, Sweden

**Keywords:** Techniques and instrumentation, Chemical engineering, Actuators, Fluidics

## Abstract

By using the temperature dependence of viscosity, we introduce a novel type of microfluidic lab-on-a-chip back pressure regulator (BPR) that can be integrated into a micro-total-analysis-system. A BPR is an important component used to gain pressure control and maintain elevated pressures in e.g. chemical extractions, synthesis, and analyses. Such applications have been limited in microfluidics, since the back pressure regularly has been attained by passive restrictors or external large-scale BPRs. Herein, an active microfluidic BPR is presented, consisting of a glass chip with integrated thin-film heaters and thermal sensors. It has no moving parts but a fluid restrictor where the flow resistance is controlled by the change of viscosity with temperature. Performance was evaluated by regulating the upstream pressure of methanol or water using a PID controller. The developed BPR has the smallest reported dead volume of 3 nL and the thermal actuation has time constants of a few seconds. The pressure regulation were reproducible with a precision in the millibar range, limited by the pressure sensor. The time constant of the pressure changes was evaluated and its dependence of the total upstream volume and the compressibility of the liquids is introduced.

The use of back pressure regulators (BPRs) can be crucial in both micro and macro fluidics. This simple, yet efficient, device widens the range of possible fluidic systems and enables solvents and mixtures that are otherwise unattainable. An ordinary pressure regulator reduces the upstream pressure to obtain a desired level at its outlet, downstream the regulator. In contrast, a BPR maintains a desired pressure upstream of the regulator. By adjusting the flow restriction and thereby the pressure drop over the BPR, the pressure at its inlet can be controlled. This allows a system with low flow restriction to be kept at elevated pressure, and ensures that it will not drop below the set level of the BPR.

Using an elevated pressure allows for higher solubility between solvents and hinders single-phase fluids to separate into more phases. It also enables heightened temperatures without the risk of gas formation. For example, in chemical synthesis, the pressure affects the solubility and reaction rate, and is important to monitor for reliable results^1,2^. For chemical extractions, high pressure and temperature are needed for pressurised liquid extractions^3^. In chemical analysis, elevated pressures are controlled to e.g. study 2-phase systems, dew points or multicomponent thermodynamics^4–6^. The stability provided by a BPR can also improve the performance of different detectors like UV spectroscopy^7^ that are commonly used in chromatography^8,9^. In microfluidic high-pressure chromatography, using elevated back pressure allows for applications in supercritical fluid chromatography^10–12^, enhanced fluidity liquid chromatography^13^ and high temperature precision chromatography^14,15^.

Today, there are several methods used for setting the back pressure. The simplest method is to use a capillary with high restriction where the length and diameter are adjusted to reach the desired pressure drop. As simple as it is, it has to be adjusted for every change in flow rate, temperature or composition.

To maintain the same pressure with different flow rates, there are mechanical BPRs that adjust the restriction of the fluid with a spring that deflects a diaphragm. When the pressure reaches the set level, the spring compresses which allows more fluid through. For smaller ones, the set level cannot be changed and separate components have to be bought for each desired pressure level. There are more complex mechanical BPRs where the set level can be changed, however, mechanical BPRs need a certain inner volume as it is crucial for their working principle.

During the last decades, much work has been put into miniaturizing fluidic systems. Miniaturization has the advantages of less sample and solvent volumes needed, less waste, and often lower costs. It is also crucial for the development of portable analysis systems that can be used for increased access to healthcare and environmental analyses in the field. Considerable work has been focused on separation techniques, synthesis, and analysis. However, there is a lack of progress in the miniaturization of surrounding inevitable components like pumps and pressure regulators^16^. To realize micro-total-analysis-system (µTAS), it would be beneficial to develop more components based on the lab-on-a-chip technology. Although several functions can be integrated into one chip, modular solutions are often preferable^17^. To avoid heat dissipation this would be the preferred method for the presented BPR.

Mechanical BPRs can have as much as 5 mL dead volume and, although several variants of smaller devices exist^18–21^, the smallest state-of-the-art BPR today has a dead volume of 5 µL^22^. However, as available components in high-performance liquid chromatography (HPLC) shrink from the microlitre scale to the nanolitre scale and below, it is of importance that associated devices follow. If a BPR is used at other positions than the outlet in an analytical system, e.g. between different detectors, the total volume downstream the column to the end of the used detector should not exceed half of the column volume, to avoid decreasing the resolution with more than 10%^23^. Hence, for microfluidic chromatography, the BPR inner volume should be at least three magnitudes smaller than what the smallest available alternatives have today. Further miniaturization of mechanical BPRs is difficult without the loss of resolution, accuracy and precision. Therefore, making such development without having performance losses requires an actuation method without moving parts.

Today, there exist no miniaturized or on-chip BPRs at the nanolitre scale that is suitable for integration with µTAS. The aim of this paper is to present such a device with results comparable to larger state-of-the-art BPRs and to provide a general understanding of the capabilities and challenges of miniaturized BPRs. The presented device incorporates a fluid restrictor where the pressure drop is regulated by thermally shifting the viscosity of the used fluid.

Temperature control is an interesting and often important issue in microfluidics. The small volumes associated are advantageous for rapid thermal shifts but can be disadvantageous in terms of stability. Hence, different approaches have been evaluated with respect to ramp time, homogeneity and temperature stability^24^. For external heating, preheated liquids have been used in separate fluidic systems running close to the microfluidic application, providing convective heating. For higher integration, chemically reacting fluids have been used for heat diffusion. In both cases, the liquids can also be used for cooling. The drawback is that the temperature changes are often slow. Joule heating and microwaves are methods that offer rapid heating and with an additional cooling system and feedback control, they can also reach sub-degree stability. By utilizing a battery, Joule heating is suitable for integration into portable lab-on-a-chip systems.

Heating has sparingly been used to regulate the pressure in supercritical fluid chromatography where the density of CO_2_ changes with temperature^25,26^. In recent work, we presented the use of viscosity changes in a thermally controlled microfluidic restrictor to regulate the composition of methanol and CO_2_, and to stabilise pressure using flow capacity, downstream of the device^17,27^. Previously, Joule heating has been used with platinum thin-films located inside the fluid channel for rapid response^17,28^. Herein, thin gold films, used for heating, have been placed in close proximity but outside the channels, to avoid unwanted chemical reactions with the metal. By that, the fluids used will only be in contact with glass, which provides excellent chemical resistance, optical transparency, and pressure tolerance. In this paper, the presented device has a higher flow restriction and is evaluated for thermal control of back pressure, i.e. the upstream pressure. Regulating back pressure, in contrast to downstream conditions, makes the volume and compressibility of the upstream system decisive to the BPR performance. The small volume in the BPR is only heated for milliseconds, and the device placed at the end of the system does not affect the temperature upstream. Its absence of moving parts makes it unique as a BPR.

## Theory

### Temperature impact on viscosity

**Figure 1:**
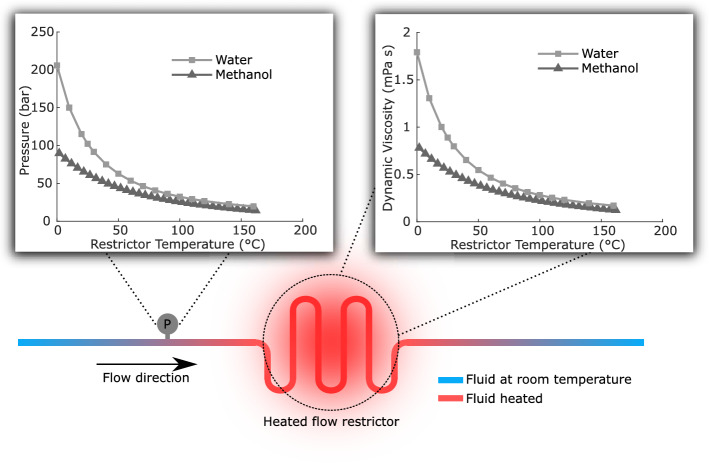
The concept of the thermally controlled BPR. The graph to the right shows the temperature dependence of viscosity for saturated liquid for water^28^ and methanol^26^. The graph to the left shows how the resulting upstream pressure depends on the temperature of the restrictor. The values are based on Eq. 2 with a flow rate of 50 µl/min and a semi-circular restrictor with the length of 10 mm and depth of 9 µm, i.e. similar to our BPR.

Regulation of back pressure can be achieved by changing the pressure drop through a restrictor. The pressure drop, ΔP, through a pipe is defined by the Hagen Poiseuille equation. By introducing the hydraulic diameter as

1 $$D_{H} = \frac{4A}{{P_{W} }}$$

where A is the cross-sectional area and P_W_ is the wetted perimeter, the equation can be written

2 $$\Delta P = \frac{8\mu LQ}{{\pi \left( {\frac{{D_{H} }}{2}} \right)^{4} }}$$

where µ is the viscosity of the fluid, L is the length of the pipe and Q is the volume flow rate. A mechanical BPR alters the hydraulic diameter by a moving diaphragm or piston. To avoid moving parts, as an alternative the viscosity can be changed by altering the temperature. The viscosity of water and methanol, and its relation to temperature are shown in Fig. 1. With a change in viscosity, the change in flow resistance will make an immediate change in flow rate. However, with compressibility, the pressure change will have a longer time constant, as explained below.

### Compressibility

Compressibility, β, is the relative change of volume, V, with pressure

3 $$\beta = - \frac{1}{V}\frac{\partial V}{{\partial P}}.$$

In a confined volume, the fluid mass storage is changing with a pressure change, where the additional fluid capacitance is

4 $$C = - \beta V.$$

In analogy with a voltage change for an electrical capacitance, the pressure will change with time, t, according to

5 $$\frac{\partial P}{{\partial t}} = \frac{Q}{C} = - \frac{Q}{\beta V}.$$

Liquids are generally considered incompressible, which is an approximation suitable for most low-pressure applications^29^. However, with rising pressure, the effects can be significant. Water and methanol have a compressibility of 46 and 125 ppm/bar, respectively. This leads to longer stabilisation times when working at high pressure. A flow system with a total volume of 100 mL and a compressibility of 100 ppm/bar can be used as an example. To raise the pressure with 10 bar, 100 µL of fluid needs to be added. If the flow rate is set to 10 µL/min and the system is closed, this would take 10 min. However, in an open system where fluid continuously exits the system, the time will be much prolonged. The added volume will be the difference between the inlet, Q_in_, and outlet, Q_out_, flow rate and, as the flow rate stabilises through the system, i.e. Q_in_ and Q_out_ equalises, the pressure rises asymptotically. This is the major contribution to the time constant in such a system.

6 $$\frac{dP}{{dt}} = \frac{{Q_{in} - Q_{out} }}{C}$$

## Method

### Chip design

As mentioned in the introduction, this BPR is a flow restrictor with resistive heaters that can change the temperature, and thereby the viscosity, of a fluid. Located downstream the introduction of an analyte or the synthesis, there is a risk in having metal resistors in direct contact with the fluid. Hence, the resistors are placed nearby, but outside, the channel. By working with channels much more shallow than wide, the hydraulic diameter becomes smaller and the flow resistance increases.

The geometrical parameters are set to enable regulation in a range of commonly used back pressures^30–32^. In this study, we exemplify the capability by flowing water and methanol, two relevant liquids for chemical analyses and synthesis^33–35^.

**Figure 2:**
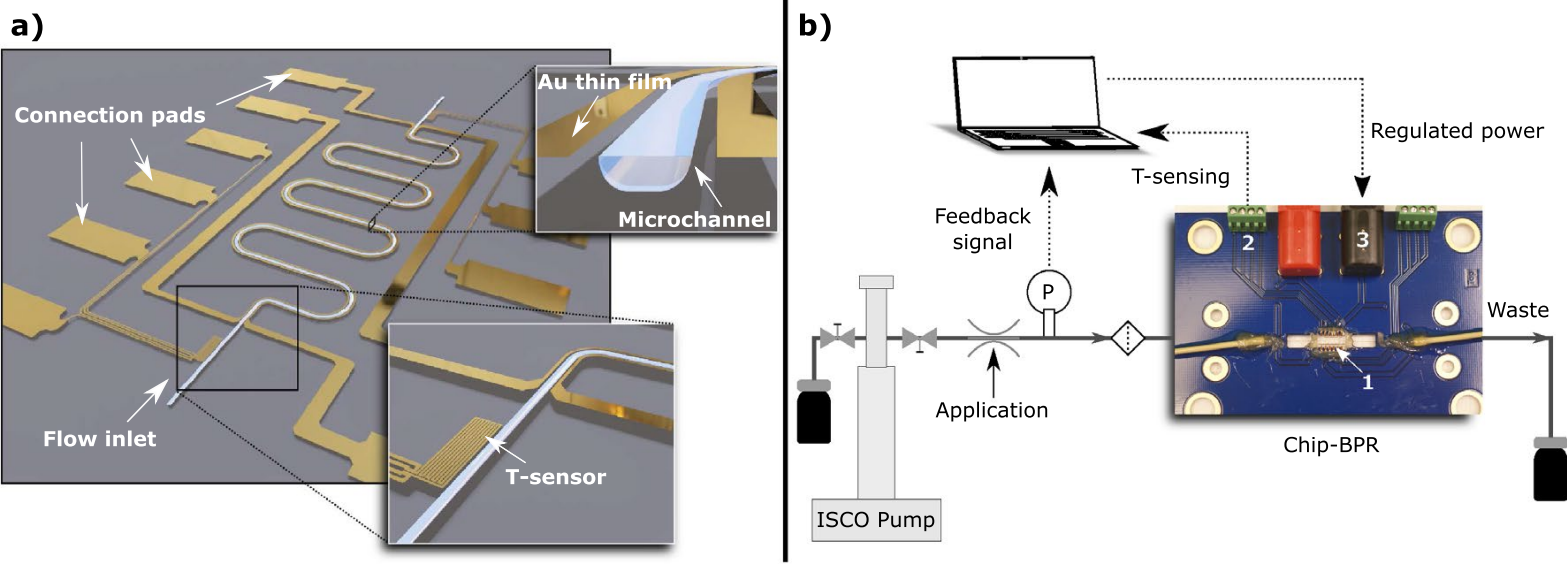
Illustration of the BPR chip design, the chip assembly and the experimental setup used for the measurements. (**a**) Sketch of the microfluidic BPR with the microchannel in light blue and thin-films in gold. The exploded image to the top-right shows the semi-circular cross-section with the thin-film heaters on both sides of the channel. The exploded image at the bottom-right shows the temperature-sensor design and position. (**b**) A schematic flow-chart of the experimental setup. The ISCO pump measures volume flow rate, pump pressure and pump volume. A photograph of the assembled chip on a PCB is included with 1. a microfluidic chip, 2. screw terminals for T-sensing, and 3. connectors for the heaters.

The chips are constructed in borosilicate glass with outer dimensions of 8 × 6 mm, resulting in 130 chips per 4-in wafer. Each chip has a 12 mm long microchannel with a semi-circular cross-section as the restrictive part of the BPR, a schematic illustration can be studied in Fig. 2a. The depth of this restrictor channel determines the pressure drop of the chip and needs to be carefully chosen depending on the range of pressure regulation that is wanted. In this paper, a depth of 8.8 µm was used. The microchannel is connected to deeper channels of 70 µm, creating an inlet and an outlet for a fluid interface to glass capillaries.

Integrated thin-films^28^ of 200 nm Au are used as heating elements and temperature sensors. Au is compatible with the fabrication process and serves as an excellent electrical contact. The heaters and sensors have interconnections to open pads on the sides of the chip for electrical connection to a printed circuit board (PCB). The temperature sensors, utilizing the change of resistivity with temperature, have a 4-point configuration with a total length of 3 mm and a width of 5 µm. The heating elements consist of two parallel-coupled resistors drawn along the channel.

Two different chip designs were made, one for temperature validation and one for back pressure regulation. The validation chip has one temperature sensor placed inside the channel and one placed next to the channel. Its design can be found in Supplementary Information. The BPR chip, Fig. 2a, has one temperature sensor at the inlet and one at the outlet of the flow restrictor, both placed next to the channel. In this chip, the heaters have a tapered geometry that starts with a width of 10 µm at the inlet and linearly increases to 27.5 µm at the middle where it remains until the outlet. The closest distance between the heaters and the channel was designed to be 10 µm.

### Fabrication

Chips were fabricated in batches by structuring thin-film Au on a glass wafer and wet etch microchannels. The wafer was then fusion bonded to a second wafer with wet etched inlet channels. A more detailed description follows.

A batch of 4-in borosilicate glass wafers (Borofloat 33, Schott) with a thickness of 1.1 mm was initially cleaned in piranha solution for 10 min, followed by RCA cleaning. Mo was then deposited as a reflective layer during lithography, a hard wet etch mask, and as a lift-off layer for the Au deposition. A pre-sputtering cleaning was performed in a mixed Phosphoric-Acetic-Nitric solution (180 H_3_PO_3_:11 HAc:11 HNO_3_:15 H_2_O) for 20 min and by an oxygen plasma etch (Plasma Processor 300, Tepla), at 1000 W for 20 min. Mo was deposited (CS730S, Von Ardenne) on both sides at 500 W for 120 s, aiming for a thickness of 250 nm.

The Au patterning was performed with UV-lithography. The Mo deposited wafer was primed with HMDS in a priming oven (Star 2000, Star) followed by spinning 1 µm resist (1813, Shipley) on both sides. The wafer was soft baked on a hot plate at 110 °C for 75 s after the first spin, and in an oven at 95 °C for 20 min after the second. The photoresist was UV-exposed and developed for 50 s.

The Mo etch mask was etched in the Phosphoric-Acetic-Nitric solution described above. Trenches in the glass were etched in buffered oxide etch (BOE 1:7, J.T.Baker) aiming for a depth of 300 nm. The thin-films were made by sputtering (Q300TD, Quorum) 30 nm Ta followed by 200 nm Au. Ta was used as the adhesive layer instead of other commonly used materials such as Ti or Cr because of a high-temperature step later in the process that causes diffusion of Cr and Ti into the gold^36–39^. The lift-off process was done in an ultrasonic acetone bath.

To form microchannels, a second lithography step with another lithography mask was made. The Mo etch mask was wet etched before the glass was etched in concentrated 49 w% HF until the set depth was reached. The second wafer, with the deeper inlet channels, was fabricated using a Mo etch mask and UV-lithography as described above. The channels were etched in HF until a depth of 70 µm was reached before removal of the etch mask.

The bonding procedure started by cleaning both wafers in Phosphoric-Acetic-Nitric solution for 20 min and oxygen plasma at 1000 W for 20 min before the activation of both wafers was done in 80 °C HNO_3_ for 15 min. The alignment was performed in a bond aligner (MA6/BA6, Karl Suss). The wafers were thermally treated at 630 °C in a furnace (Micro TF-6, Koyo Lindberg) for 6 h with a ramp up and ramp down of 1 °C/min. Finally, the bonded wafer was diced (DAD 361, Disco) into chips.

### Assembly

A chip was assembled by surface mounting its electrical pads onto a PCB with silver epoxy (CW2400, CircuitWorks). The PCBs were designed (KiCad) and fabricated (PCBWay) to fit the chips, a photo of the assembly can be found in Fig. 2b.

To gain fluidic access, glass capillaries with 105 µm outer diameter and 40 µm inner diameter (88224, Polymicro Technologies) were thermally pulled (P-1000, Sutter Instrument) to form a pointy end to fit into the 70 µm deep inlets, and glued to the chip with a two-component epoxy (EPO-TEK 730, GA Lindberg). PEEK tubing (PEEK 1/16, Upchurch Scientific) were glued onto the glass capillaries. As a final step, 4 mm connectors (CLIFF) for heaters, and screw terminals (282834-4, TE Connectivity AMP Connectors) for temperature sensors were soldered onto the PCB.

### Experimental setup

The equipment used in the measurements were a high-pressure pump (100 DM, ISCO Teledyne), a pressure sensor (33X, Keller), and steel tubing to connect the components to the assembled chip. In addition, a power supply (QL355TP, TTi) for heating and a DAQ (34970A, Agilent) for temperature sensing were connected to the PCB. Sensing and control were performed from a MATLAB script, making it possible to log the back pressure, inlet temperature, outlet temperature, pump flow rate, pump volume, and power, over time. A schematic overview of the experimental setup is illustrated in Fig. 2b. To regulate the back pressure, the script includes a PID regulator that controls the output power. To allow for rapid thermal actuation, the assembled chip was mounted on a water-cooled table with cooling paste.

### Measurements

To estimate the heat distribution on the chip, a theoretical model was made with finite element analysis (Comsol Multiphysics 5.5, Comsol) where the flow rate of water was set to 50 µL/min with *Laminar flow* and the heater voltage was set to 10 V. A 1 mm thick layer of cooling paste was added to the model with one side in contact with the glass chip and a set temperature of 8 °C on the other side. One model was made with uniform resistors and one was made with the self-compensating tapered resistors. The resistive heating was simulated with *Electric currents in shells* and added as a *Boundary heat source* in *Heat transfer in Solids and Liquids*. The multiphysics *Nonisothermal flow* was used to combine the physics.

Pressure tests of the chips were experimentally performed using methanol at room temperature. First, the flow through the chip was checked. Then, the outlet was plugged with PEEK fittings and the chip was pressurised to 10 bar, to see if the chip held tight at a constant pressure. The pressure was then increased linearly by 0.11 bar s^−1^ up to 100 bar, and then accelerated to 0.19 bar s^−1^ until leakage was observed.

The temperature sensors were calibrated inside an oven (Binder FD53, Skafte Medlab) where the resistances in both temperature sensors and the temperature in a thermocouple (type K) were measured. The oven was set to 100 °C until the resistances in the temperature sensors had stabilised. It was then switched off, and measurements were made until the oven reached room temperature.

To evaluate the difference between having temperature sensors next to the channel, and inside the channel, temperature measurements were made on the validation chip. To measure the temperature at the inlet, the fluid was introduced from the left in the figure shown in Supplementary Information. Later, the fluid was introduced from the right, to measure the temperature at the outlet. The voltage was altered with five-minute intervals.

To show the concept and how larger pressure windows can be achieved with the BPR chip, methanol or water was flowed through the chip with a controlled flow rate until the pressure was stabilised. A set voltage was then applied until a new pressure level was reached, without any regulations. Then, the voltage was turned off and the pressure was measured until it returned to its previous level.

The reproducibility and the precision of the PID-regulated BPR were evaluated by regulating the back pressure between 90 and 80 bar. Measurements were made on water or methanol. With a PID control written in MATLAB, the applied voltage was regulated to achieve the set pressure. An upper limit was set to 12 V, as it is commonly used in batteries. The time constants were calculated as the time required to reach 63.2% of the set pressure change, i.e. 86.32 and 83.68 bar for raising and falling pressure respectively. The mean pressure and standard deviation were calculated starting 5 min after the set pressure change, to let the pressure stabilise.

To evaluate how the pump volume affects the time constants, the experiment was repeated several times as the volume in the pump decreased.

## Results

### Simulations

**Figure 3:**
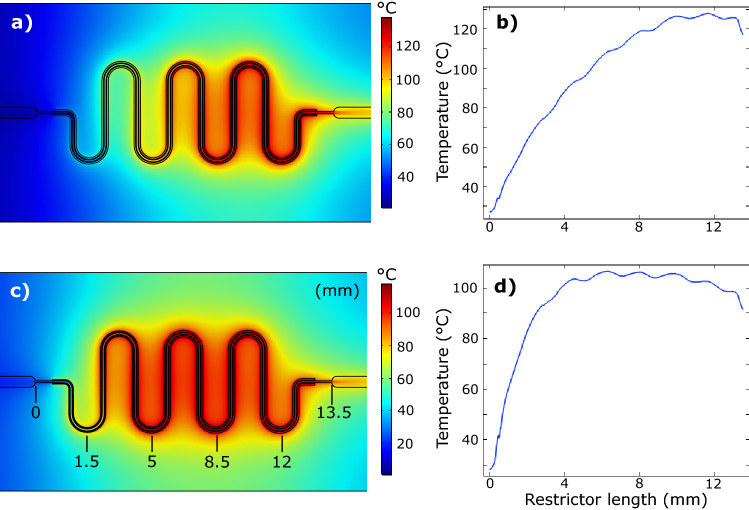
Temperature simulations with water flowing at 50 µl/min and an applied voltage of 10 V to the heaters, producing a power of 1.3 and 1.2 mW for the even-width and the tapered resistor design, respectively. The images show a cross section of the chip at the glass-glass interface. (**a**) Temperature distribution with even-width heaters and (**b**) corresponding temperature along the flow channel. (**c**) Temperature distribution with tapered heaters and (**d**) corresponding temperature along the flow channel.

Simulation results of the heat distribution in the chip with different resistor designs are shown in Fig. 3. For the even-width resistor, the temperature gradually increases along the channel and reaches a maximum temperature of 128 °C. For the tapered geometry, the thermal distribution is more even along the channel with a maximum temperature of 106 °C. The resulting pressure drop is similar, with 72.5 bar for the even-width and 72.6 bar for the tapered heater design.

### Chip characterisation

A pressure drop through the BPR chip was experimentally measured to 70 bar, using methanol at 20 °C and 40 µL/min, corresponding to a channel depth of 8.8 µm. The width of the channel was 28 µm and the total inner volume of the restrictor channel was 3 nL. The precision in the bonding alignment was within 6 µm. An applied voltage of 5 V through the heaters corresponded to a power output of approximately 0.27 W. The chip could withstand 148 bar before leakage was observed through the thin-film trenches.

**Figure 4:**
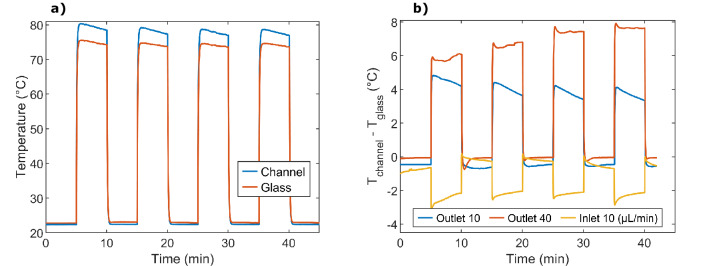
Comparative temperature measurements from the integrated temperature sensors in the validation chip. (**a**) Temperature at the flow outlet inside the channel (blue) and in the glass next to the channel (red). The voltage was alternated between 0 and 10 V and the flowrate was 10 µL/min. (**b**) Differences between the temperature inside, and next to, the channel at the outlet (blue) and the inlet (yellow), for a flowrate of 10 µL/min. Differences at the outlet for a flowrate of 40 µL/min (red).

Time constants of the thermal actuation changed with heating power, flow rate, fluid medium and cooling temperature. A comparison of the measured temperature inside and next to the channel in the validation chip is shown in Fig. 4a. The time constants for rising temperature are 4.0 and 4.2 s, and for falling temperature, 1.7 and 1.9 s, for measurements inside and next to the channel, respectively. The difference between the absolute values in the measured temperatures is shown in Fig. 4b.

### Characterisation of back pressure regulation

**Figure 5:**
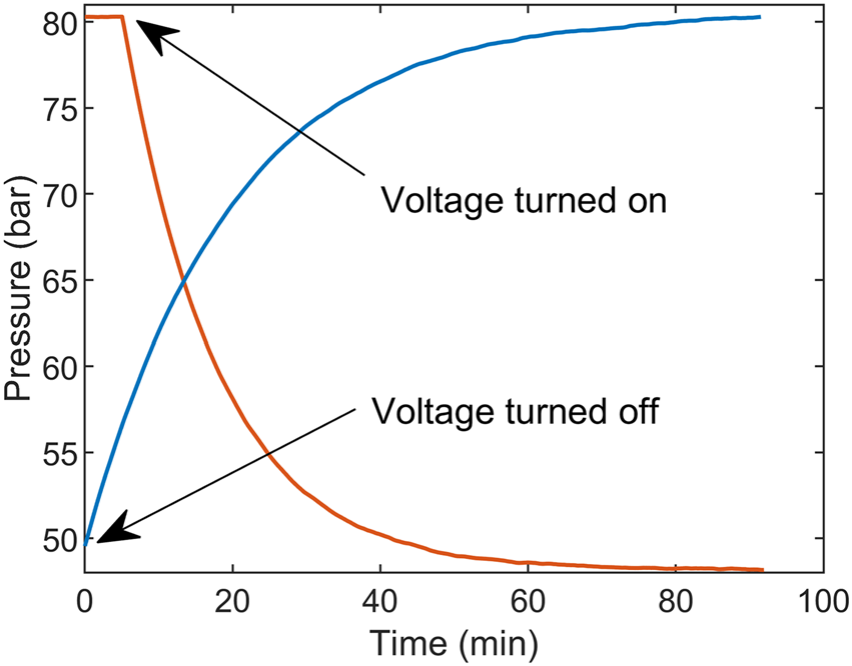
Back pressure regulation of water flowing at 18 µL/min, without PID regulation. A voltage of 5 V was applied to heat the restrictor and reduce the pressure. When the voltage is turned off, the pressure returns to its original level.

At a voltage of 10 V, the maximum span of the regulator was 70% of the maximum pressure when using methanol. In Fig. 5, water pressure drops from 80 to 48 bar (40% of the span) at a voltage of 5 V. The time constants were 17.8 and 19.3 min, when decreasing and increasing the pressure respectively.

**Figure 6:**
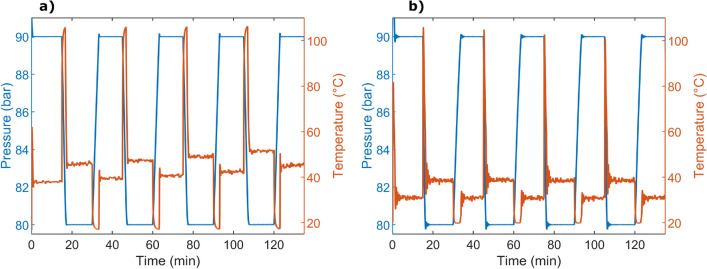
Pressure regulation between 80 and 90 bar of (**a**) water at 25 µL/min and (**b**) methanol at 40 µL/min. Included in the graphs is the temperature change causing the pressure change.

The results from shifting the back pressure between 80 and 90 bar using PID regulation are shown in Fig. 6. In the measurements with water, the flow rate was 25 µL/min, and the volume in the pump was 60 mL resulting in a time constant of 1.3 min. The mean pressures were 90 and 80 bar with standard deviations of 2 respectively 3 mbar. For long-time experiments with water at elevated temperature and pressure, corrosion was seen in the channel. Methanol had a flow rate of 40 µL/min, and with a pump volume of 15 mL, the time constant became 0.6 min. The mean pressures were 90 and 80 bar with standard deviations of 9 and 6 mbar, respectively. Here, the standard deviation of the temperature was 0.5 °C and there was no significant difference between the pressures in each cycle. The pressure sensor had a precision of 0.01% F.S. (30 mbar), and an accuracy of 0.05% F.S. (150 mbar). In the measurements, the accuracy was below 1 ppm and hence, the pressure sensor was the limiting factor for both the precision and accuracy.

**Figure 7:**
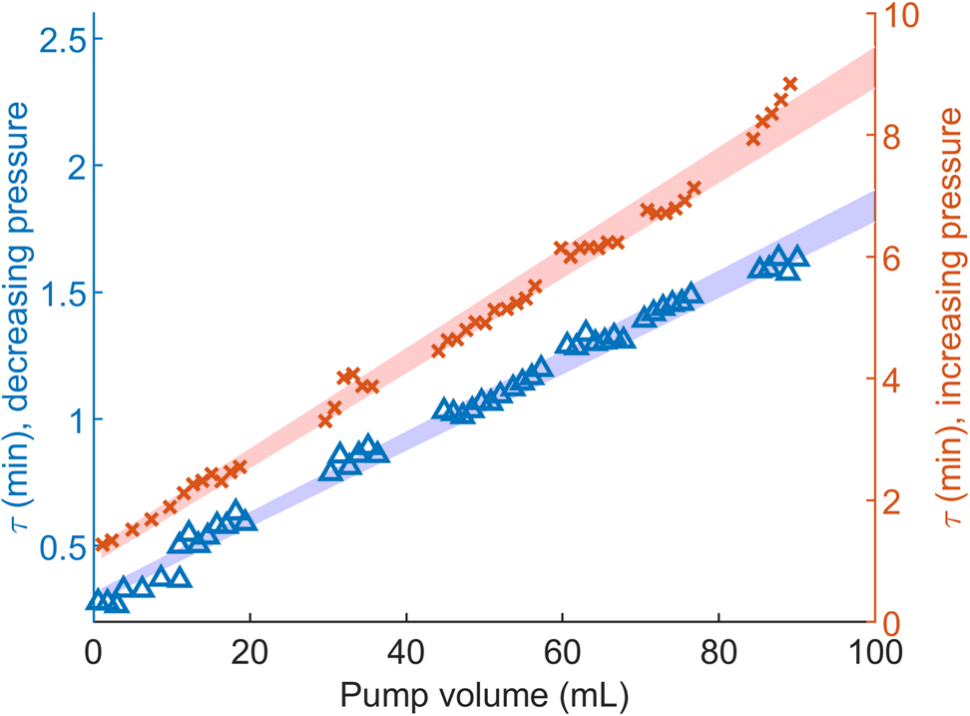
Time constants of the pressure altering between 80 and 90 bar, plotted over pump volume with a flow rate of 40 µL/min methanol. The red and blue shaded areas represent a 95% confidence interval of the regression line.

The pressure regulation with methanol was repeated with various pump volumes, and the resulting time constants are shown in Fig. 7. At the lowest volume, the time constants when increasing and decreasing pressure were 1.3 and 0.3 min, respectively. At the highest volume of 90 mL, they were 8.8 and 1.6 min, respectively.

## Discussion

The presented microfluidic BPR has characteristics comparable to larger-scale state-of-the-art devices but with a dead volume that is three magnitudes smaller than the smallest commercially available alternative. The miniaturized dimensions lead to rapid thermal actuation, enabling the use of glass, a material with limited thermal conductivity. The lack of moving parts simplifies the fabrication and with the small chip dimensions of 8 × 6 mm, 130 units could be made of a 4-in wafer. This enables components with lower fabrication and material costs. The use of the chemically resistant glass allows the use of analytes that otherwise react with the metals often found in today’s BPRs. Moreover, the pressure-resistant material allows for high-pressure applications.

The BPR showed reliability with reproducible results over time, Fig. 6. The cooling system efficiently removed excessive heat as no drift was seen. Unlike a passive restrictor, the pressure levels could be maintained with different flow rates and fluid mediums. The PID controller enables stability over time as it compensates for changes in the system, making the BPR suitable for long-time experiments. The precision and accuracy of the presented device are in the same range as the best commercial BPRs^18,40,41^, and are limited by the pressure sensor.

In the simulation, Fig. 3, the temperature profiles along the channel differ for the two heater designs, while the resulting pressure drops were the same. The maximum temperature for the tapered design is 22 °C lower than the even-width design, making it beneficial for temperature-sensitive fluids. It also enables higher powers, i.e. larger pressure changes, to be used without the risk of gas formation.

The time constants of the thermal actuation, Fig. 4, are in the same order as previously reported Joule heating^24^. In the validation chip, the temperature sensor next to the channel showed a 0.2 s higher time constant than the sensor inside the channel. Compared to measuring temperature on the chip’s outer surface, the distance for the heat transfer is minimal which results in a fast response.

In a previous work^17^, heaters had been placed inside the fluid channel for faster heating response. In this work, the heaters were placed next to the channel to avoid unwanted electrochemical reactions and the risk of leakage through the electrodes. Even though the heaters were not in contact with the fluid, and the glass has a low thermal conductivity^12^, the temperature sensors responded immediately. Comparing the seconds-long heating and the minutes-long pressure regulation, it can be concluded that placing the heaters inside or with a µm-proximity to the channel will only result in a negligible difference in the pressure regulation time constant. This is possible thanks to the small thermal mass of 0.1 cm^3^ and the short distance of 10 µm between the channel and the heaters. Consequently, this heating method is suitable for not only back pressure regulation but also for other microfluidic applications requiring rapid thermal control and stability.

The previous use of platinum required up to 30 V for sufficient actuation. Herein, gold was used which has four times higher conductivity than platinum and hence, a lower voltage was needed for the same power. This is beneficial for future portable systems where a 9 or 12 V battery could be used as power supply. With a battery, pulse-width modulation could be used for regulation.

The time constants for the pressure regulation depend on the used power and the size of the pressure shift. The impact of the total volume of the system, including the pump volume, is less intuitive. Although it is well known that fluidic systems need time to stabilise, the compressibility is often neglected for liquids. The effect the compressibility has on the time constant was explained in the Theory section and demonstrated experimentally by plotting the time constants toward the pump volume, Fig. 7. However, the volume of the total system also includes the pressure sensor, tubing, and the BPR chip. The effect of the volume can also be seen in Fig. 6; when the pressure is set to 80 bar, the temperature quickly rises to above 100 °C. With time, the height of these peaks gets lower, corresponding to the lowered pump volume. With smaller total volumes, the time constants would have been further reduced until limited by the time constants of the thermal actuation.

As the time constant highly depends on the system, specific values are not presented for commercial BPRs and a direct comparison is not possible. Still, when comparing the method used here with mechanical BPRs, both change the flow restriction to adjust the flow rate and thereby, the pressure. In addition, the spring compression in a mechanical BPR creates a void volume for the fluid to fill. This instant volume change results in a rapid response. Hence, with larger volumes, larger pressure changes can be made with short time constants. Consequently, when a smaller dead volume is wanted, the time constant will be longer regardless of the technique used. It is however, standard procedure to let a fluidic system stabilise for a couple of minutes before any experiment is started. For µTAS, further miniaturizing of mechanical BPRs could be possible but with the cost of reduced resolution, precision and accuracy. Wear, clogging and complicated fabrication are also associated problems with moving parts in microfluidics. The presented on-chip BPR uses voltage to regulate the temperature, which bypasses these problems. Miniaturization of electronic components is successfully implemented in today’s society and the PCB and power regulation could be further optimized to fit into specific portable systems.

The relation between the time constants for heating and cooling differs in Figs. 4 and 7. The cooling was performed by a water-cooled table with a circulating flow where the temperature and circulation speed could be set. If the ramp time of either heating or cooling, i.e. decreasing or increasing the pressure, is crucial for a given application this could be adjusted. Generally, an equal relation should be used for optimal PID regulation. Here, this was difficult to achieve as the required cooling depends on the flow rate of the fluid in the chip and the temperature range of the heating. In further work, another cooling system that could be integrated into the regulation system would be beneficial. Then, even faster temperature changes could be achieved by turning off the cooling when heating starts and vice versa. A more compact cooling system would also be necessary to miniaturize the whole system as the cooling bath stands for the bulkiest part. It is important that, for any chosen cooling system, it has the capacity of removing enough excess heat to avoid thermal drift.

The result in Fig. 5 shows how the pressure is changing when a constant voltage is applied. The temperature and the viscosity of the fluid change immediately. A lower viscosity and an elevated upstream pressure rapidly increase the flow rate in the chip, Q_out_ in Eq. (6), causing a pressure drop. As the pressure decreases, the increased flow rate will follow, slowing down the rate of the pressure drop. The closer the pressure comes to its new stabilisation point, the closer the flow rate comes to its starting point, as described in Eq. (6).

The maximum pressure change achievable depends on the used power and fluid composition. By applying 10 V, the pressure of methanol was changed by 70%. Allowing higher voltage, and thereby power, would further increase the range. The limit is reached when the density of the fluid decreases more than its viscosity. In Eq. (2), Q is the volumetric flow rate, and if density decreases, the volume will increase, resulting in a higher pressure. For other flow rates or pressure levels, the channel dimension can be adjusted. With 40 µL/min, the fluid stays in the channel for 4 ms. For adjusted parameters, the linear velocity and thus, the resulting time in the channel should be considered. A deeper channel can be used for a lower pressure drop and, since that will result in a lower linear velocity, the channel can be shortened to minimize the volume. To increase the pressure drop, a shallower channel can be used, which will increase the linear velocity. If this results in insufficient heating time, or risk of clogging, a higher resistance can instead be achieved with a longer channel.

The difference in temperature, shown in Fig. 4b, depends on the flow rate and the position of the sensor. At the inlet, the sensor inside the channel is colder by its direct contact with the fluid entering at room temperature. With higher flow rate, the difference to the sensor next to the channel increases. At the outlet, the water-cooled table and the ambient temperature provide cooling, while the now heated fluid contributes to a higher temperature inside the channel than next to it. The difference, as well as the absolute temperature, increases with an increasing flow rate. This is explained by the higher flow rate decreasing the overall temperature at the beginning of the chip, which lowers the resistance of the resistive heaters. With a set voltage and lower resistance, the current increases, which was seen in both experiments and simulations. This results in an increased power, leading to a higher absolute temperature at the end of the chip.

In Fig. 4b, for the experiment with 40 µL/min, one can see that the difference in temperature inside, and next to, the channel increases for each cycle. This is caused by an unstable system where the pressure increases with time. The slope within each cycle for the measurements with 10 µL/min is caused by the temperature in the chip that is stabilising with heat dissipation.

For the water in Fig. 6a, the temperature sensor showed peaks of 106 °C. As seen in the simulations, Fig. 3, the sensor is not placed in the hottest area and the maximum temperature will reach even higher values. Unfortunately, deionised water is corrosive to borosilicate glass at temperatures above 100 °C, and corrosion could be observed in the channels after several high-temperature runs. Hence, working with water requires an adjusted voltage limit to avoid these temperatures, alternatively another chip material, e.g. silicon. However, the stabilised temperature needed to maintain 80 or 90 bar only differs with 5 °C, while the peak temperatures are a matter of getting quicker pressure changes. For temperature-sensitive experiments, it should be noted that a BPR is usually placed at the end of the system. The small volume of the chip only heats a fraction of the fluid for a very short time, and the temperature of an upstream experiment is not affected. If a temperature-sensitive fluid shall pass the BPR, a limitation can be added to the PID regulator. Either the maximum applied voltage can be restrained, or a maximum temperature could be set by adding the integrated temperature sensors to the feedback system.

## Conclusion

A microfluidic BPR has been developed to enable high-precision back pressure regulation without adding a large dead volume into the system. It is actively regulating the back pressure using the viscosity shift with temperature. The device can be used in many microfluidic applications e.g. pressurised liquid extractions, chemical synthesis, and chemical analysis such as various liquid chromatography applications. The presented BPR has, to our knowledge, the smallest dead volume presented, with an internal volume of 3 nL. The device has no moving parts and the fluids are only in contact with glass. The maximum pressure tolerance was 148 bar, and with a customized PID regulation, the BPR reached a precision and accuracy as low as that of the feedback pressure sensor. The pressure span depends on the fluids’ viscosity profile and the absolute pressure is set by the restrictor design and the flow rates. A relative span of 70% of the maximum pressure was achieved for methanol. The BPR heats only a fraction of the fluid and does not affect the temperature upstream. If a sensitive fluid shall pass the BPR e.g. when the BPR is placed between detectors, a limit should be added to the PID regulation. An upper power limitation should also be added when working with pressurised deionised water as it is corrosive to glass when temperature exceeds 100 °C. The BPR is a proof-of-concept device and the future outlook contains improved pressure tolerance and understanding of how to regulate compressible and multicomponent fluids. Finally, portability can be achieved by coupling the BPR with other lab-on-a-chip modules and integrating it into a battery-powered µTAS.

## Supplementary Information

Supplementary Information.
