# Supplementary material for: Thermally controlled microfluidic back pressure regulator
Source: Sci Rep. 2022 Jan 12;12:569. doi: 10.1038/s41598-021-04320-6 (PMC8755753; doi:10.1038/s41598-021-04320-6)
Supplement: Supplementary file 1 — Supplementary Information. [file 41598_2021_4320_MOESM1_ESM.pdf]

# Supplementary Information

## Thermally Controlled Microfluidic Back Pressure Regulator

**Karolina Svensson†*, Simon Södergren*, Klas Hjort†**

**Microsystems Technology Division, Centre of Natural Hazard and Disaster Science (CNDS), Uppsala University, Box 35, 751 03 Uppsala, Sweden**

*** Shared first authors**

**† Corresponding authors:** Karolina.svensson@angstrom.uu.se, klas.hjort@angstrom.uu.se

## Validation chip design

To validate a design where the temperature sensors are placed next to the channel, a validation chip was made with one sensor inside the channel and one close to the channel. The total number of temperature sensors was constrained to two for each chip due to the limited space for connection pads. With this design, the temperature at the inlet could be evaluated by flowing from left to right, and the temperature at the outlet could be evaluated by flowing from right to left, Figure 1.

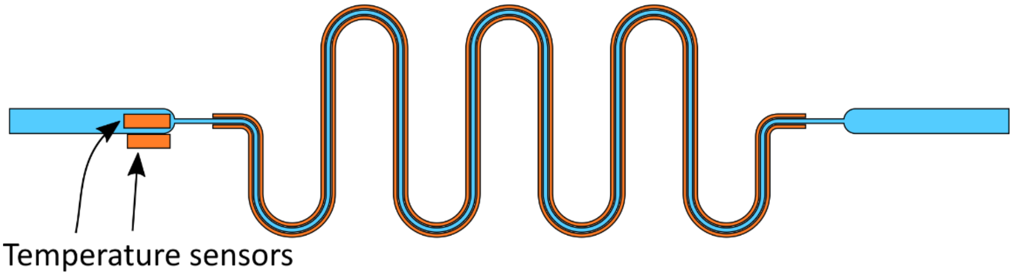


**Figure 1:** Schematic illustration of the validation chip, used for comparative temperature measurements inside and outside the microfluidic channel. The blue part is the microfluidic channel and the orange parts are the Au thin-films used for heating and temperature sensing. The restrictive part of the microchannel is 13 mm long. The thin-films used as heating elements along the restrictive channel have an even-width of 24 µm. The temperature sensors have a total length of 3 mm and a width of 5 µm.
